# Intersection of DNA Repair Pathways and the Immune Landscape Identifies PD-L2 as a Prognostic Marker in Epithelial Ovarian Cancer

**DOI:** 10.3390/cancers13081972

**Published:** 2021-04-20

**Authors:** Samantha Batman, Koji Matsuo, Paulette Mhawech-Fauceglia, Elizabeth Munro, Mercedes Weisenberger, Allison Allen, Sonali Joshi, Hiroko Machida, Shinya Matsuzaki, Tatjana Bozanovic, Tanja Pejovic

**Affiliations:** 1 Division of Gynecologic Oncology, Department of Obstetrics and Gynecology, Oregon Health & Science University, Portland, OR 97239-3098, USA; batman@ohsu.edu (S.B.); munro@ohsu.edu (E.M.); willimer@ohsu.edu (M.W.); landstro@ohsu.edu (A.A.); joshiso@ohsu.edu (S.J.); 2 Division of Gynecologic Oncology, Department of Obstetrics and Gynecology, University of Southern California, Los Angeles, CA 90033, USA; koji.matsuo@med.usc.edu (K.M.); pfauceglia@auroradx.com (P.M.-F.); zacky_s@gyne.med.osaka-u.ac.jp (S.M.); 3 Norris Comprehensive Cancer Center, University of Southern California, Los Angeles, CA 90033, USA; 4 Aurora Diagnostic Center of Excellence in Gynecologic Pathology, Austin, TX 78758, USA; 5 Tokai University School of Medicine, Tokyo 151-8677, Japan; hiroko.machida@tokai.ac.jp; 6 Department of Obstetrics and Gynecology, University of Belgrade, 11000 Belgrade, Serbia; tab145712@yahoo.com

**Keywords:** ovarian cancer, PD-L2, survival, PARP

## Abstract

**Simple Summary:**

Here, we examined the interaction between DNA repair proteins and immune biomarkers and their association with survival in 181 cases of epithelial ovarian carcinoma (EOC). We used a panel of 12 antibodies for immunocytochemistry staining of tissue microarray (TMA) consisting of 181 cases. Applying standard statistical methods, we detected that PD-L2 expression was associated with decreased survival in ovarian cancer. This is the first demonstration that increased expression of PD-L2 may serve as a marker for decreased progression-free survival (PFS). Therefore, further investigation into PD-L2 based immunotherapy as a strategy to treat ovarian cancer is warranted.

**Abstract:**

Background: Targeting DNA repair and immune checkpoint pathways has been the focus of multiple clinical trials. In this study, we explore the association between DNA repair proteins, immune response markers, and clinical outcome in women with EOC. Methods: Immunohistochemical analysis of TMA with 181 EOC samples was used to determine expression levels for DNA repair proteins (PARP, PTEN, p53, H2Ax, FANCD2, and ATM) and immune-markers (CD4, CD8, CD68, PD-L2, PD-L1, and FOXP3). Biomarker expression was correlated to clinical data. Prognostic discriminatory ability was assessed per the combination of biomarkers. Results: Tumor immunity biomarkers correlated with HRD biomarkers. High PD-L2 was significantly associated with high expression of CD8 (*r* = 0.18), CD68 (*r* = 0.17), and FOXp3 (*r* = 0.16) (all, *p* < 0.05). In a multivariate analysis, PD-L2 (hazard ratio (HR) 1.89), PARP (HR 1.75), and PTEN (HR 1.96) expressions were independently associated with decreased progression-free survival (PFS), whereas PD-L1 (HR 0.49) and CD4 (HR 0.67) were associated with improved PFS (all, *p* < 0.05). In 15 biomarker combinations, six combinations exhibited a discriminatory ability of >20% for the 4.5-year PFS rate, with four based on PD-L2 (PARP, PTEN, CD4, and PD-L1, 20.5–30.0%). Conclusions: Increased PD-L2 expression is a prognostic marker of decreased survival in EOC. Interaction between tumor DNA repair and microenvironment determines tumor progression and survival.

## 1. Introduction

Most women with ovarian cancer present with advanced disease at diagnosis and despite an initial response to treatment, the majority experience recurrence and die of chemo-resistant disease. Given the poor response of recurrent disease to traditional chemotherapy, there is a need to develop alternative treatment strategies. Two potential therapeutic targets, i.e., the DNA repair pathway and the immune checkpoint pathway, are being vigorously investigated and both are in clinical trials in ovarian and other cancers.

Exploiting the vulnerability of DNA repair deficient ovarian cancer, PARP inhibitors olaparib, niraparib, and rucaparib have been granted FDA approval for primary or secondary maintenance or treatment of recurrent ovarian cancer [1,2]. Meanwhile, the programmed cell death 1 (PD-1) and its ligand (PD-L1) pathway, which inhibits anticancer response, have been investigated in many tumor types. PD-1 expression in ovarian cancer has been associated with unfavorable prognosis [3]. In preclinical models, disruption of the PD-1 pathway has been shown to enhance tumor immune response [4], suggesting that PD-1/PD-L1 has a role in ovarian cancer treatment. While PD-1 is a transmembrane receptor expressed at the cell surface of T cells, B cells, monocytes, NK cells, and dendritic cells [5], inducible expression of PD-L2 is mainly through Th2-associated cytokines, on the surface of macrophages, dendritic cells, and other immune and non-immune cells [6]. PD-L1 and PD-L2 compete for binding to PD-1, and PD-L2 has two–six-fold higher affinity than PD-L1 [7]. PD-L2 is generally expressed at lower levels than PD-L1 [8], but PD-L2 expression is significantly higher during Th2 responses [9].

Targeting both DNA repair and immune checkpoint pathways has emerged as a concept based on the unique characteristic of DNA repair deficient tumors as genetically unstable and characterized by production of tumor-specific neoantigens. This concept has been recently investigated in clinical trials and a recent molecular subclassification of samples from a trial of niraparib and pembrolizumab in platinum resistant ovarian cancer yielded important results [10]. Nonetheless, it is still largely unknown which biomarkers predict tumor response to immune treatment or combined therapy.

Here, we investigate the correlations between selected DNA repair proteins, the immune checkpoint pathway, and survival in patients with sporadic epithelial ovarian cancer.

## 2. Materials and Methods

### 2.1. Study Population

All patients underwent surgical staging or debulking for epithelial ovarian, fallopian tubal, or primary peritoneal carcinoma. All pathology specimens were collected and reviewed, and tumors were classified according to the World Health Organization (WHO) criteria [11]. The medical records of the patients were retrospectively reviewed under an approved Institutional Review Board protocol (Oregon Health and Sciences IRB 921) in accordance with relevant guidelines and regulations, and informed consent was obtained as required by the IRB. The review included outpatient and inpatient treatment, including surgery and chemotherapy. Survival information for overall survival (OS) and progression-free survival (PFS) were also collected.

### 2.2. Tissue Microarrays

Ovarian cancer tissue microarrays (TMAs) were constructed using paraffin-embedded archival tissue from 181 eligible patients with epithelial ovarian cancer, as described previously [12,13]. Hematoxylin-eosin (HE) staining was used to select morphologically representative regions and core biopsies were sampled from 3 distinct areas of each tumor to account for tumor heterogeneity. Triplicate 0.6 mm cores were punched from the individual donor formalin-fixed, paraffin-embedded blocks, and transferred to the TMA paraffin-embedded receiver blocks. One section from each TMA was stained with H&E to confirm the presence of the tumor by light microscopy [13].

### 2.3. Immunohistochemistry Assay and Scoring Criteria

Immunohistochemistry was performed, as previously described [13]. Sections (4 μm) were deparaffinized and pretreated in citrate buffer pH 6.0 for 20 min, cooled 20 min, and incubated 10 min at ambient temperature in 3% H_2_O_2_ to quench endogenous peroxidase activity. Blocking was performed using serum-free protein block, (Dakocytomation, Carpenteria, CA, USA) for 30 min. Sections were incubated with antibodies to ATM, FANCD2, PTEN, H2AX, PARP, p53, and CD4, CD8, CD68, FOXP3, PD-L1, and PD-L2 and conditions are summarized in Table 1 [14].

### 2.4. Study Definition

In this study, various cutoffs of immunohistochemistry intensity for each biomarker were examined for outcome measures assessed by log-rank test (Tables S1 and S2), and the cutoff point exhibiting the largest statistical value was chosen for each cutoff. For PFS, the cutoffs were: 0 vs. 1–3 (H2Ax and PD-L2), 0–1 vs. 2–3 (PARP, PTEN, and PD-L1), and 0–2 vs. 3 (ATM and FANC). For CD4, CD8, CD68, and FOXp3 results, the results among those that scored >0 were trichotomized as 1–33 percentile, 34–66 percentile, and 67–100 percentile. The log-rank value was determined in each cutoff for each biomarker, and the largest statistical value was used for the cutoff as follows: none vs. 1–100 percentile (CD4), none/1–33 percentile vs. 34–100 percentile (CD68 and FOXp3), and none/1–66 percentile vs. 67–100 percentile (CD8).

The rationale of this cutoff analysis was to provide a mechanistic approach to detect a more predictive cutoff for survival as compared with our prior analysis where the biomarker expressions were only dichotomized as 0–1 vs. 2–3. In this way, we identified 2 biomarkers (ATM and FANCD2) that a higher cutoff (0–2 vs. 3) predicted PFS better as compared with the historical cutoff (0–1 vs. 2–3). Likewise, we identified two biomarkers (H2Ax and PD-L2) that a lower cutoff (0 vs. 1–3) predicted PFS better vs. the historical cutoff. For consistency purposes, a similar methodology was undertaken for cell counts in our study.

The age cutoff was 70, per prior studies. High-grade serous ovarian cancer was defined as grade 2–3 serous carcinoma, as described before [15]. PFS was defined as the time interval between the initial surgery and the first recurrence/progression of disease, or death from ovarian cancer. OS was the interval between surgery and death from all cause. Data were censored at the last follow-up for patients without these survival events.

### 2.5. Statistical Analyses

The primary step of the analysis was to examine the correlation of examined biomarkers and clinical and pathological factors. The secondary step of the analysis was to assess the prognostic impact of the examined biomarkers.

Continuous variables were expressed as a mean (±SD). Categorical and ordinal variables were expressed as a number (%). Spearman’s correlation coefficient was used to examine the statistical significance of biomarkers expressed with correlation coefficient value. For survival analysis, the Kaplan–Meier method was used to construct the survival curves, and statistical difference between the curves was assessed by a log-rank test in univariate analysis. A Cox proportional hazard regression model was fitted to identify the independent prognostic markers for survival (PFS or OS) in the multivariate analysis. In this study, covariates with *p*-value of less than 0.20 in univariate analysis were chosen for the model construction. This relatively liberal cutoff of the covariate selection was due to the small sample size in our study. The effect size for survival outcome measures was expressed by adjusted-hazard ratio (HR) with corresponding 95% confidence interval (CI).

For a sensitivity analysis, the effects of biomarker combination on survival were examined. Specifically, among the significant biomarkers for PFS on multivariate analysis, combination patterns of any two markers were assessed. Then, among four patterns in each biomarker combination of the two, the absolute difference between the lowest and highest survival rate at the specific point estimate, namely the discriminatory ability for survival rate, was computed. A *p*-value of less than 0.05 was considered to be statistically significant. All analyses were based on the two-tailed hypothesis. IBM SPSS Statistics, version 25.0 (Armonk, NY, USA) was used for the analysis.

## 3. Results

### 3.1. Study Population and Cohort Characteristics

There were 181 cases examined for analysis. Patient’s characteristics at baseline are summarized in Table 2. The mean age of the study population was 61.6 years, and the majority of the patients had ovarian cancer (75.1%), grade 3 tumors (86.2%), and stage III–IV disease (90.0%). The most common histology type was high-grade serous (*n* = 141) in this study population. Complete cytoreduction was recorded in only about one-third of cases (34.1%). All patients received platinum-based first line chemotherapy.

### 3.2. Biomarker Correlations

**Figure 1:**
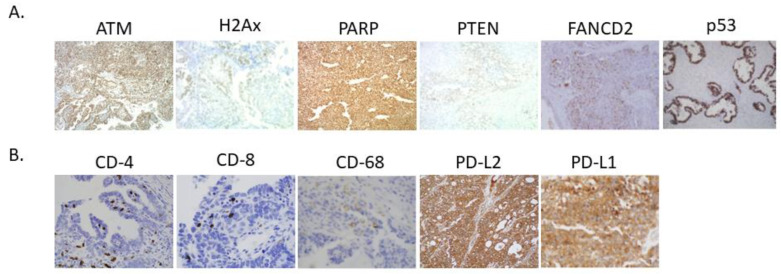
Expression of DNA repair and immune checkpoint markers in epithelial ovarian tumors. Representative immunohistochemistry staining of (**A**) DNA repair (ATM, H2Ax, PARP, PTEN, FANCD2, and p53) and (**B**) immune markers (CD-4, CD-8, CD-68, PD-L2, and PD-L1) in a tissue microarray with clinically annotated ovarian tumor tissue.

DNA repair marker (ATM, H2Ax, PARP, PTEN, FANCD2, and p53) and immune marker expression (CD-4, CD-8, CD-68, PD-L2, and PD-L1) was observed in the ovarian cancer tissue array (Figure 1 and Figure S1). Correlation among selected DNA repair deficiency biomarkers and tumor microenvironment immune markers were examined (Table 3). Overall, the biomarkers representing tumor immunity correlated well to the biomarkers representing DNA repair. Specifically, strong correlations (*r* > 0.20) were observed between CD-4 and PARP (*r* = 0.24), CD8 and ATM (*r* = 0.27), PD-L1 and PARP (*r* = 0.59), PD-L1 and ATM (*r* = 0.32), and PD-L1 and FANCD2 (*r* = 0.37) (all, *p* < 0.05). Of interest, PD-L2 expression was significantly associated with CD8, CD68, and FOXp3 (all, *p* < 0.05). PD-L2 and PARP were not associated (*p* = 0.34). Similar findings were seen in 141 cases of high-grade serous ovarian cancer (Table S3). PD-L2 expression was significantly associated with CD68 expression (*r* = 0.17, *p* = 0.048).

### 3.3. Survival Statistics

The median follow-up time of the patients who were alive at the last visit was 4.7 years (interquartile range 2.9–6.7). Nearly 90% of the study population had recurrence, progression, or death from ovarian cancer (87.7%). There were 140 deaths recorded during the follow-up. The median estimated PFS and OS for the whole cohort was 1.8 (95% CI 1.4–2.1) and 3.4 (95% CI 2.9–3.8) years, respectively. The 4.5-year PFS rate of the entire cohort was 19.7% (95% CI 14.0–26.1). Of note, 4.5-year survival estimates were based on the median follow-up of censored cases.

### 3.4. Prognostic Biomarkers

Independent characteristics for PFS were examined in a multivariate analysis (Table 4). Three biomarkers were found to be prognostic for decreased PFS (PARP, PTEN, and PD-L2). Specifically, moderate-strong expression of PARP was associated with decreased PFS as compared with negative-mild expression (4.5-year rates, 16.1% vs. 25.6%, HR 1.75, 95% CI 1.19–2.59); moderate-strong PTEN expression was associated with decreased PFS as compared with negative-mild expression (10.4% vs. 21.1%, HR 1.96, 95% CI 1.13–3.41); and any PD-L2 expression was associated with decreased PFS as compared with no expression (18.4% vs. 32.8%, HR 1.89, 95% CI 1.01–3.52). In contrast, expression of PD-L1 remained an independent prognostic factor associated with improved PFS (4.5-year rates 22.0% versus 17.4%, HR 0.49, 95% CI 0.32–0.73). Moreover, higher CD4 and CD8 cell counts were associated with improved PFS, i.e., 4.5-year rates for any positive vs. negative CD4 cell counts, 22.9% vs. 12.5%, HR 0.67, 95% CI 0.45–0.99 and 3rd quartile cell counts versus negative/1st–2nd quartile cell counts, 24.9% vs. 17.6%, HR 0.68, 95% CI 0.45–1.04.

Independent characteristics for OS were assessed in multivariate analysis (Table 5). Among the tested biomarkers, only CD8 cell counts remained statistically significant for OS. Specifically, any positivity for CD8 cell counts was associated with improved OS as compared with negative cell count (4.5-year rates 34.0% vs. 14.8%, HR 0.60, 95% CI 0.36–0.99). For both outcome measures for survival, complete cytoreduction status was independently associated with both PFS (4.5-year rates 32.0% vs. 13.8%, HR 0.64, 95% CI 0.43–0.95) and OS (39.9% vs. 25.8%, HR 0.61, 95% CI 0.41–0.92). Higher stage was associated with decreased PFS in this study (4.5-year rates 15.8% vs. 66.7%, HR 2.28, 95% CI 1.02–5.10).

### 3.5. Biomarker Combination Patterns and Survival Outcome

**Figure 2:**
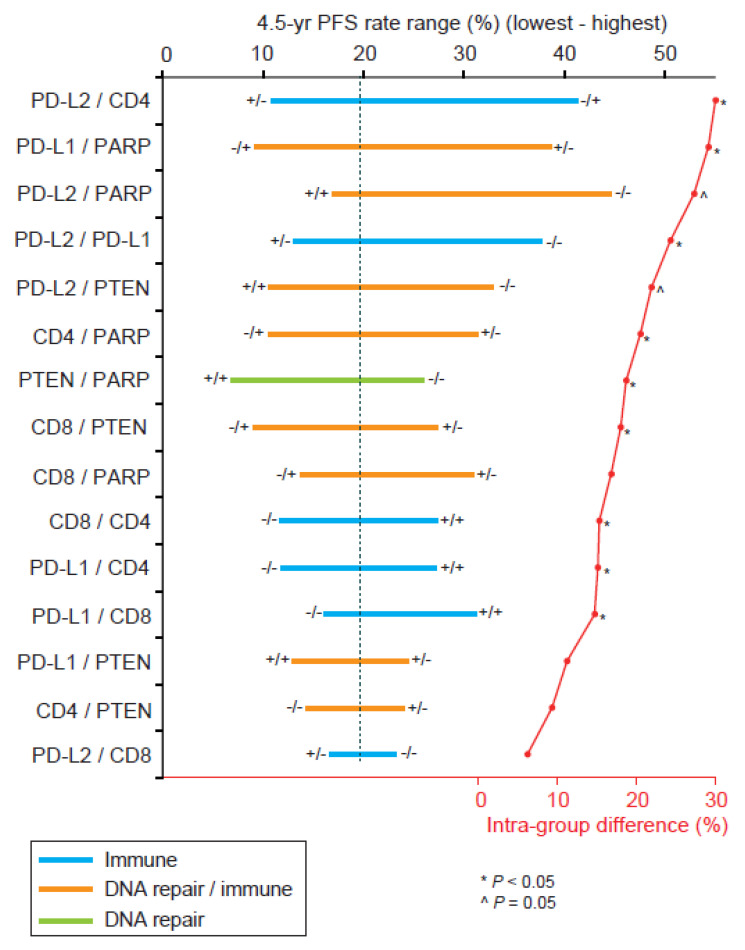
Discriminatory ability of progression-free survival based on protein microarray results. Among 6 biomarkers identified in multivariate analysis (Table 3), combination patterns of any two biomarkers were assessed (total 15 patterns). In each pattern, biomarker expression was assessed as negative/negative (−/−), negative/positive (−/+), positive/negative (+/−), and positive/positive (+/+). The cutoff definition of negative and positive results is displayed in Table 3. Then, the 4.5-year progression-free survival (PFS) rates were computed, and discriminatory ability defined as the difference between the lowest and highest rates was estimated and shown by red dots and line (displayed as the secondary axis for intra-group difference). A log-rank test was used for *p*-values (lowest vs. highest). Subgroups with fewer than ten cases were not interpreted for analysis due to insufficient number. Bars represent the highest PFS rate (right side) and the lowest PFS rate (left side) in each biomarker combination. The dashed line indicates the 4.5-year PFS rate of 19.7% for the entire study cohort.

**Figure 3:**
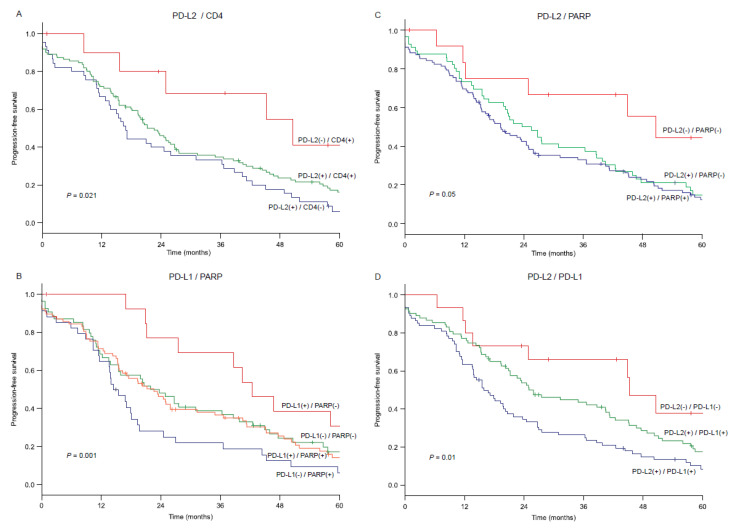
Survival curves for progression-free survival based on combination patterns. Survival curves are constructed with the Kaplan–Meier method per the combination pattern of biomarkers. (**A**) PD-L2 and CD4; (**B**) PD-L1 and PARP; (**C**) PD-L2 and PARP; (**D**) PD-L2 and PD-L1. In Figure panels (**A**,**C**,**D**), subgroups with small number (<9) were censored due to futility. Log-rank test for *p*-values.

According to the results of the PFS analysis (Table 3), the combination pattern of six biomarkers were examined (i.e., PARP, PTEN, PD-L1, PD-L2, CD4, and CD8). Any two biomarkers were chosen, and a total of 15 combinations were assessed for PFS (Figure 2). Of those, six combinations exhibited a discriminatory ability of >20% point for the 4.5-year PFS rate, of which four combinations were based on PD-L2 (PARP, PTEN, CD4, and PD-L1). The large discriminatory ability for the 4.5-year PFS rate was seen in the combination of PD-L2 and CD4 (30.0%, *p* = 0.021, Figure 3A) followed by the combination of PD-L1 and PARP (29.1%, *p* < 0.01, Figure 3B), the combination of PD-L2 and PARP (27.3%, *p* = 0.05, Figure 3C), and the combination of PD-L2 and PD-L1 (24.3%, *p* = 0.01, Figure 3D).

Among the 15 combination patterns, there were two patterns with an estimated 4.5-year PFS rate that exceeded 40% (Figure 2). The highest one was the combination of negative PD-L2 and negative PARP expressions (44.4%, Figure 3C), followed by negative PD-L2 and positive CD4 cell counts (41.1%, Figure 3A). This was followed by positive PD-L1 and negative PARP expressions (38.5%, Figure 3B) and negative PD-L2 and negative PD-L1 expression (37.7%, Figure 3D). In contrast, there were three patterns that the estimated 4.5-year PFS rate was <10% (Figure 2). These included negative PD-L1 and positive PARP expressions (9.4%, Figure 3B), negative CD8 cell count and positive PTEN expression (9.2%), and positive PTEN and positive PARP expressions (7.1%).

## 4. Discussion

Inhibitors of immune checkpoints have recently shown therapeutic success in several tumor types, yet a significant number of patients do not respond to this class of drugs. To date, no immune checkpoint inhibitor (ICI) has been approved by the U.S. Food and Drug Administration for the treatment of patients with ovarian cancer. In order to successfully apply ICIs to the treatment of ovarian cancer, it is essential to define molecular and other predictors of response to these agents. Several recent observations have suggested that tumors with defects in homologous recombination (HR) pathway display genomic instability, high tumor mutational burden, and an increased repertoire of neoantigens, are more immunogenic, leading to adaptive upregulation of PD-L1 by tumor and sensitization to ICI [16]. Another important observation is that PARP inhibition leads to an accumulation of cytosolic double-stranded DNA, and thereby activates the cytoplasmic DNA sensor cyclic guanine monophosphate/adenosine monophosphate synthase/stimulator of interferon genes (cGAS/STING) [17]. This pathway triggers activation of a signaling cascade connecting cGAS to signal transducers, including STING and TBK1, and eventually to transcription factors (mainly IRF3 and nuclear factor-κB) that collectively induce a type I interferon response, and thereby turn “cold” non-T cell-inflamed tumors into “hot” T cell-inflamed tumors. The same principle molecular mechanism was the rationale for the promising antitumor activity seen in a recent trial (TOPACIO/KEYNOTE-162), using niraparib and pembrolizumab in platinum resistant ovarian cancer [18]. Furthermore, a recent large collaborative initiative utilized the tumor samples from this same trial to analyze immune predictors of tumor response. The mutational signature reflecting the combination of defective DNA repair and a positive immune score was found to be associated with an improved outcome. In contrast, the absence of either defective DNA repair or positive immune score was associated with no response to combination treatment. In addition, single-cell spatial analysis revealed prominent interactions of CD8 + T-cells with PD-L1 + macrophages and PD-L1 + tumor cells as mechanistic determinants of response [19].

In light of these significant contributions for elucidating the biomarkers of ICI sensitivity, we set out to determine the connections between immune and DNA markers in a cohort of 181 patients with sporadic ovarian cancer. As the BRCA1 immunocytochemical staining was overwhelmingly and repeatedly positive in the majority of our samples, we selected six other DNA repair protein markers, based on our prior experience [14]. As expected, the current cross analysis of DNA repair markers and immune markers revealed positive correlations between several markers from the two groups. Of particular interest is that PD-L2 expression was associated with overexpression of CD8, CD68, and FOXp3 suggesting that PD-L2 may induce response of different classes of T cells as well as macrophages.

Survival outcomes depend on the cancer itself and also on the tumor microenvironment of HGSOC which is rich in immune cells and tumor-associated macrophages [20]. An abundance of cytotoxic CD8^+^ T cells as well as CD4^+^ in ovarian cancer is a prognostic indicator of greater PFS and OS [21,22,23,24,25]. In our series, we show similar results with high numbers of CD4 and CD8 cell counts associated with prolonged PFS. In the multivariate analysis, the expressions of PARP, PTEN, and PD-L2 were associated with decrease survival. PARP expression is thought to increase the capacity of cells to repair DNA breaks accounting for decreased survival. PTEN is traditionally considered to be a tumor suppressor and its loss is associated with poor prognosis. However, its expression in HGSOC is heterogeneous and effects of PTEN may vary depending on its cytoplasmic or nuclear localization which was not considered in this study. Interestingly, in our study PD-L1 was found to be an independent prognostic factor associated with improved PFS. While several studies have indeed suggested that PD-L1 is a good prognostic (and therapeutic) target in ovarian cancer, the results are inconsistent and many other studies have found no correlation between PD-L1 expression by ICH and PFS or OS in ovarian cancer [26].

A novel finding in this study is the correlation of PD-L2 expression with decreased PFS. PD-1 ligands, PD-L1 and PD-L2, bind to inhibitory molecule PD-1 and together play a key role in the induction of immune tolerance in the tumor microenvironment. While the PD-1/PD-L1 pathway (pembrolizumab, nivolumab/avelumab, atezolumab) has been extensively studied in cancer, including ovarian, the role of PD-L2 in cancer and immunity is less clear and the therapeutic role for PD-L2 inhibition has not been broadly explored. Nevertheless, our results corroborate work of few other studies [9]. Prior studies have found an impaired survival in patients with PD-L2 expressing tumors, yet this trend has rarely reached statistical significance, and in the majority of studies, PD-L2 was expressed in only a minority of cases [27,28,29]. In a cohort of 70 patients with ovarian cancer, the majority of the cases were negative or weakly positive and although PD-L2 expression was correlated with an impaired survival, this did not reach statistical significance [30].

These results highlight the need to explore the potential therapeutic role of PD-L2 as a single factor or in combination. Yearley et al. [31] described a positive response of PD-L2+ /PD-L1 tumors to pembrolizumab, suggesting that the PD-1-PDL-2 axis may function independently of PD-L1 [31,32]. Moreover, Ahmad et al. characterized a CD8 and CD4 T cell PD-L2 specific T cells and showed that they do not cross react with PD-L1-specific T cells [32]. They postulated that PD-L2 specific T cells activated by vaccination can exhibit anticancer immunity by directly killing target cells, or by indirectly altering the microenvironment through cytokine release [32].

According to our combinatorial analysis, the lack of PD-L2 expression combined with the lack of PARP expression were the strongest predictor of increased survival. Additionally, positive PD-L2 expression together with either CD4 or PARP expression was associated with increased PFS and points to known synergistic mechanisms that can be further explored. All combinatorial patterns suggest that exploring these leads may confirm the known effects of these combinatorial pairs and possibly reveal novel mechanisms of PD-L1 and PD-L2 interactions.

Our study has several limitations. First, we only selected to test a limited number of DNA repair proteins based on their known prognostic relevance in ovarian cancer and availability of reliable antibodies, yet the list is not comprehensive. Second, this is a descriptive study based on immunocytochemistry and though we tested several antibodies to determine the optimal antibody, the results should be validated by additional techniques in additional cohorts. In addition, BRCA1 antibody had suboptimal performance while BRCA2 antibody was not tested because of its known heterogenous immunochemical results. Finally, the utility of biomarker cutoffs proposed in the analysis has not be validated in an independent cohort and warrants further validation study.

## 5. Conclusions

In conclusion, we report a previously unrecognized association of PD-L2 with decreased survival in ovarian cancer. To the best of our knowledge, this is the first demonstration that increased expression of PD-L2 may serve as a marker for decreased progression-free survival, providing justification for further investigation into the PD1/PD-L2 axis and PD-L2 based immunotherapy as a strategy to treat ovarian cancer.

## Figures and Tables

**Table 1:** Immunohistochemistry summary.

Antibody	Company	Primary Antibody	Dilution	Antigen Retrieval	Incubation	Positive Control
PARP	Abcam	Monoclonal	1/25	Citrate buffer, 20 min steamer	1 h	Breast carcinoma
PTEN	Millipore	Monoclonal	1/100	Citrate buffer, 20 min microwave	2 h	Endometrial cancer
ATM	Abcam	Monoclonal	1/50	TRIS, 40 min steamer	1 h	Normal testis
FANCD2	Epitomics	Monoclonal	1/100	Citrate buffer, 20 min microwave	1 h	Breast carcinoma
H2AX	Bethyl Lab	Monoclonal	1/100	Citrate buffer, 20 min microwave	1 h	Ovarian cancer
p53	Novocastra	Monoclonal	1/50	Citrate buffer, 40 min microwave	1 h	Ovarian cancer
CD4	Agilent	Monoclonal	ready to use	Automated	30 min	Tonsil
CD8	Agilent	Monoclonal	ready to use	Automated	30 min	Tosnil
CD68	Agilent	Monoclonal	1/3000	Automated	30 min	Lymph node
FOXP3	Biolegend	Polyclonal	1/20	Tris buffer	30 min	Lymph node
PDL1	Lifespan	Polyclonal	1/250	Alkaline buffer, 20 min	30 min	Tonsil
PDL2	Atlas	Polyclonal	1/100	Citrate buffer, 40 min microwave	1 h	Tonsil

Immunostaining (the intensity of positive tumor cells) was reviewed using conventional light microscopy and scored by a board-certified gynecologic pathologist (P.M.F.). For PARP, ATM, H2AX, PTEN, FANCD2, PDL1, and PDL2, staining intensity was categorized as 0, 1+ (light brown), 2+ (moderate brown), or 3+ (dark brown). For CD4, CD8, CD68, and FOXP3 interpretation, only intraepithelial T cell (CD4, CD8, and FOXP3) and intraepithelial tumor-associated macrophages (CD68) were evaluated. For this interpretation, 10 independent areas evaluating all 3 cores were done. Lost, severely damaged, or cores without sufficient tumor cellularity were not evaluated. The reviewer was blinded to clinical data. Ovarian cancer cases were excluded from statistical analysis if triplicate cores were unavailable for analysis secondary to inadequate cancer tissue or poor quality of the specimen [13,14].

**Table 2:** Patient demographics.

Characteristics	*n* = 181
Age (yrs) *	61.6 (±12.1)
<70	(71.4%)
≥70	(28.6%)
Primary site	
Ovary	136 (75.1%)
Fallopian	1 (0.6%)
Peritoneal	44 (24.3%)
Cancer Stage *	
I	8 (4.4%)
II	10 (5.6%)
III	138 (76.7%)
IV	24 (13.3%)
Histology type	
Serous *	147 (81.2%)
Clear cell	8 (4.4%)
Endometrioid	9 (5.0%)
Mucinous	5 (2.8%)
Mixed	9 (5.0%)
Carcinosarcoma	3 (1.7%)
Grade tumor differentiation	
1	10 (5.5%)
2	15 (8.3%)
3	156 (86.2%)
Complete resection	
No	118 (65.9%)
Yes	61 (34.1%)
Progression of disease	
No	24 (13.3%)
Yes **	157 (87.7%)
Death due to disease	
No	30 (16.8%)
Yes	149 (83.2%)

Mean (± SD) or number (%) is shown. * 1 missing. * Serous histology includes 141 (77.9%) of high-grade histology and 6 (3.3%) of low-grade histology. ** Including 68 cases of recurrence or progression and 89 cases of persistent disease with ultimate succumbing to death. Total number may not be 181 due to missing information. Median follow-up time was 37.3 months.

**Table 3:** Correlation analysis for biomarkers.

	No.	p53		PARP		ATM		H2Ax		PTEN		FANCD2		CD4		CD8		CD68		FOXp3		PD-L1		PD-L2	
p53	173			0.13		0.08		0.02		0.01		0.09		0.18		0.07		0.18		0.08		0.13		0.07	
	
PARP	181	0.08				0.41		0.04		0.06		0.40		0.24		0.07		0.07		0.09		0.59		0.07	
	
ATM	173	0.31		<0.001				0.22		0.12		0.32		0.002		0.27		0.15		0.13		0.32		0.04	
	
H2Ax	178	0.76		0.57		0.004				0.15		0.05		0.04		0.02		0.02		0.05		0.01		0.03	
	
PTEN	179	0.89		0.44		0.13		0.044				0.02		0.08		0.01		0.09		0.05		0.14		0.04	
	
FANCD2	179	0.25		<0.001		<0.001		0.50		0.80				0.09		0.07		0.08		0.03		0.37		0.07	
	
CD4	179	0.02		0.001		0.98		0.62		0.31		0.21				0.45		0.49		0.46		0.08		0.01	
	
CD8	176	0.40		0.33		<0.001		0.80		0.90		0.38		<0.001				0.49		0.55		0.12		0.18	
	
CD68	173	0.02		0.34		0.046		0.79		0.23		0.30		<0.001		<0.001				0.57		0.10		0.17	
	
FOXp3	177	0.32		0.26		0.10		0.51		0.51		0.71		<0.001		<0.001		<0.001				0.05		0.16	
	
PD-L1	180	0.09		<0.001		<0.001		0.94		0.055		<0.001		0.29		0.11		0.20		0.53				0.10	
	
PD-L2	178	0.38		0.34		0.64		0.73		0.58		0.33		0.95		0.02		0.03		0.03		0.20			
	

**Table 4:** Independent factors for progression-free survival (*n* = 181, whole cohort).

Characteristic	4.5-yr (%)	Adjusted HR (95% CI)	*p*-Value
Stage			
I–II	66.7%	1	
III–IV	15.8%	2.28 (1.02–5.10)	**0.046**
Cytoreduction			
Residual	13.8%	1	
Complete	32.0%	0.64 (0.43–0.95)	**0.026**
PARP			
0/1+	25.6%	1	
2+/3+	16.1%	1.75 (1.19–2.59)	**0.005**
PTEN			
0/1+	21.1%	1	
2+/3+	10.4%	1.96 (1.13–3.41)	**0.017**
FANC			
0/1+/2+	17.3%	1	
3+	25.9%	0.82 (0.55–1.24)	0.350
PD-L1			
0/1+	17.4%	1	
2+/3+	22.0%	0.49 (0.32–0.73)	**<0.001**
PD-L2			
0	32.8%	1	
1+/2+/3+	18.4%	1.89 (1.01–3.52)	**0.046**
CD4			
0	12.5%	1	
1+/2+/3+	22.9%	0.67 (0.45–0.99)	**0.042**
CD8			
0/1+/2+	17.6%	1	
3+	24.9%	0.68 (0.45–1.04)	0.073

The point estimate of 4.5 years for the survival rate was based on the median follow-up of the censored cases in the cohort. Cutoffs of each biomarkers are shown in Table S1. Only the factors exhibited *p* < 0.20 on univariate analysis were entered in the model as above. A Log-rank test was used for the univariate analysis. A Cox proportional hazard regression model was used with conditional backward method for multivariate analysis. Significant *p*-values are emboldened. Abbreviations: 4.5-yr (%), 4.5-year progression-free survival; HR, hazard ratio; and CI, confidence interval.

**Table 5:** Independent factors for overall survival (*n* = 181, whole cohort).

Characteristic	4.5-yr (%)	Adjusted-HR (95%CI)	*p*-Value
Age (years)			
<70	34.3%	1	
≥70	24.1%	1.40 (0.96–2.05)	0.079
Stage			
I–II	69.3%	1	
III–IV	28.4%	2.12 (0.90–5.01)	0.086
Cytoreduction			
Residual	25.8%	1	
Complete	39.9%	0.61 (0.41–0.92)	**0.019**
H2Ax			
0	65.9%	1	
1+/2+/3+	28.9%	1.64 (0.82–3.30)	0.163
PD-L1			
0/1+	27.3%	1	
2+/3+	35.8%	0.83 (0.59–1.16)	0.273
CD8			
0	14.8%	1	
1+/2+/3+	34.0%	0.60 (0.36–0.99)	**0.045**
FOXp3			
0/1+	28.9%	1	
2+/3+	35.6%	0.74 (0.51–1.08)	0.115

## Data Availability

Data is contained within the article and supplementary material.

## Supplementary Materials

The following are available online at https://www.mdpi.com/article/10.3390/cancers13081972/s1, Figure S1: Weak/negative expression of DNA repair and immune checkpoint markers in epithelial ovarian tumor, Table S1: Cutoff values for biomarkers (progression-free survival), Table S2: Cutoff values for biomarkers (overall survival), Table S3. Correlation for pathological factors among high-grade serous carcinoma (*n* = 141).
