# Supplementary material for: Intersection of DNA Repair Pathways and the Immune Landscape Identifies PD-L2 as a Prognostic Marker in Epithelial Ovarian Cancer
Source: Cancers (Basel). 2021 Apr 20;13(8):1972. doi: 10.3390/cancers13081972 (PMC8073346; doi:10.3390/cancers13081972)
Supplement: Supplementary file 1 [file cancers-13-01972-s001.zip › cancers-1176752-supplementary.pdf]

Supplementary Materials: Intersection of DNA Repair Pathways and the Immune Landscape Identifies PD-L2 as a Prognostic Marker in Epithelial Ovarian Cancer

Samantha Batman, Koji Matsuo, Paulette Mhawech-Fauceglia, Elizabeth Munro, Mercedes Weisenberger, Allison Allen, Sonali Joshi, Hiroko Machida, Shinya Matsuzaki, Tatjana Bozanovic and Tanja Pejovic

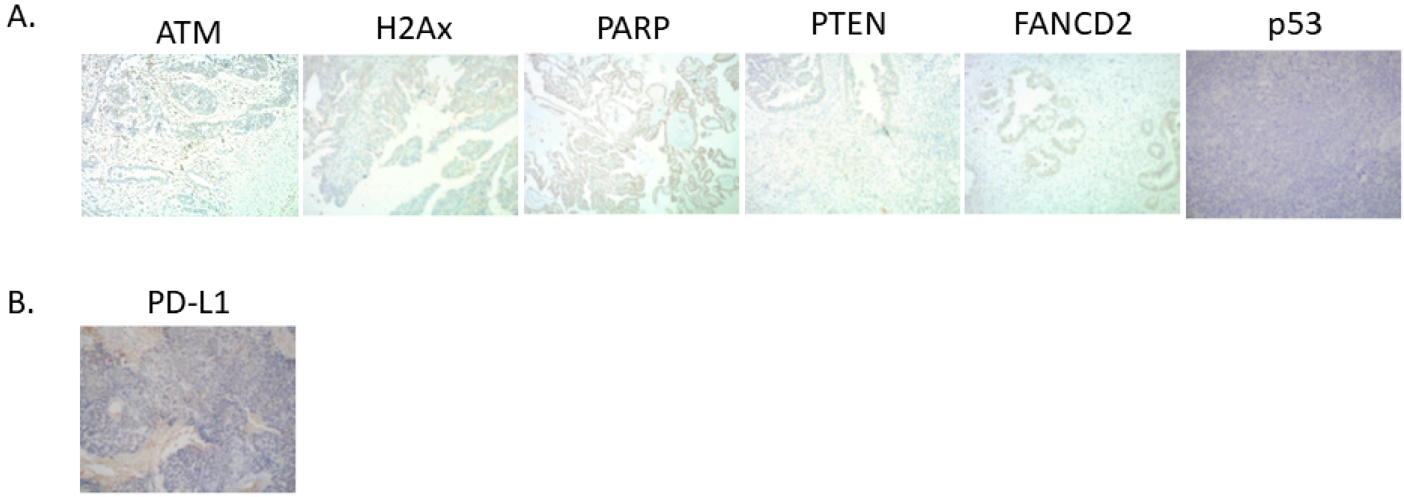

Figure S1. Weak/negative expression of DNA repair and Immune checkpoint markers in epithelial ovarian tumors. Representative weak/negative immunohistochemistry staining of (A) DNA repair (ATM, H2Ax, PARP, PTEN, FANCD2, p53) and (B) immune markers (PD-L1) in a tissue microarray with clinically annotated ovarian tumor tissue.

Table S1. Cutoff values for biomarkers (progression-free survival).

IHC Cutoff	0 vs. 1+/2+/3+	0/1+ vs. 2+/3+	0/1+/2+ vs. 3+	Final Cutoff Chosen for Survival Analysis
PARP	Log-rank 0.165	Log-rank 1.947	Log-rank 0.284	0/1+ vs. 2+/3+
ATM	Log-rank 0.100	Log-rank 0.445	Log-rank 1.233	0/1+/2+ vs. 3+
H2Ax	Log-rank 1.246	Log-rank 0.202	Log-rank 1.057	0 vs. /1+/2+/3+
PTEN	Log-rank 0.025	Log-rank 1.974	Log-rank 0.161	0/1+ vs. 2+/3+
FANC	Log-rank 0.061	Log-rank 0.040	Log-rank 1.797	0/1+/2+ vs. 3+
PD-L1	Log-rank 0.114	Log-rank 2.625	Log-rank 1.281	0/1+ vs. 2+/3+
PD-L2	Log-rank 2.675	Log-rank 1.796	Log-rank 0.055	0 vs. 1+/2+/3+

Cell Counts	0 vs. 1st-T/2nd-T/3rd-T	0/1st-T vs. 2nd-T/3rd-T	0/1st-T/2nd-T vs. 3rd-T	Final Cutoff Chosen for Survival Analysis
CD4	Log-rank 2.864	Log-rank 0.100	Log-rank 0.021	0 vs. 1st-T/2nd-T/3rd-T
CD8	Log-rank 0.974	Log-rank 1.469	Log-rank 2.362	0/1st-T/2nd-T vs. 3rd-T
CD68	Log-rank 0.087	Log-rank 0.383	Log-rank 0.177	0/1st-T vs. 2nd-T/3rd-T
FOXp3	Log-rank 0.275	Log-rank 1.571	Log-rank 1.272	0/1st-T vs. 2nd-T/3rd-T

For the biomarkers that scored via immunohistochemistry (PARP, ATM, H2Ax, PTEN, FANC, PD-L1, and PD-L2) as 0, 1+, 2+, or 3+, we have modeled the tested biomarkers as the following 3 patterns: 0 vs. 1+/2+/3+; 0/1+ vs. 2+/3+; and 0, 1+, 2+ vs. 3+. In each cutoff, log-rank test was performed for progression-free survival, and the statistical values are displayed in the table. Among the 3 cutoffs tested, the cutoff demonstrating the largest statistical value was chosen for the further survival analysis. For the biomarkers that scored as cell counts (CD4, CD8, CD68, and FOXp3), the cell counts among expressed were trisected as 1-33%ile (1st-T), 34-66%ile (2nd-T), and 67-100%ile (3rd-T). We have modeled the tested biomarkers as the following 3 patterns: 0 vs. 1st-T/2nd-T/3rd-T; 0/1st-T vs. 2nd-T/3rd-T; and 0/1st-T/2nd-T vs. 3rd-T. In each cutoff, Log-rank test was performed for progression-free survival, and the statistical values are displayed in the table. Among the 3 cutoffs tested, the cutoff demonstrating the largest statistical value was chosen for the further survival analysis.

Table S2. Cutoff values for biomarkers (overall survival).

IHC Cutoff	0 vs. 1+/2+/3+	0/1+ vs. 2+/3+	0/1+/2+ vs. 3+	Final Cutoff Chosen for Survival Analysis
PARP	Log-rank 0.031	Log-rank 0.075	Log-rank 0.021	0/1+ vs. 2+/3+
ATM	Log-rank 0.071	Log-rank 0.545	Log-rank 0.333	0/1+ vs. 2+/3+
H2Ax	Log-rank 2.144	Log-rank 0.103	Log-rank 0.684	0 vs. 1+/2+/3+
PTEN	Log-rank 0.001	Log-rank 0.385	Log-rank 0.168	0/1+ vs. 2+/3+
FANC	Log-rank 1.093	Log-rank 0.153	Log-rank 1.905	0/1+/2+ vs. 3+
PD-L1	Log-rank 0.775	Log-rank 1.974	Log-rank 1.698	0/1+ vs. 2+/3+
PD-L2	Log-rank 0.004	Log-rank 0.025	Log-rank 1.059	0 vs. 1+/2+/3+

Cell Counts	0 vs. 1st-T/2nd-T/3rd-T	0/1st-T vs. 2nd-T/3rd-T	0/1st-T/2nd-T vs. 3rd-T	Final Cutoff Chosen for Survival Analysis
CD4	Log-rank 0.512	Log-rank 0.008	Log-rank 0.016	0 vs. 1st-T/2nd-T/3rd-T
CD8	Log-rank 3.951	Log-rank 1.057	Log-rank 1.521	0 vs. 1st-T/2nd-T/3rd-T
CD68	Log-rank 0.000	Log-rank 0.171	Log-rank 0.690	0/1st-T/2nd-T vs. 3rd-T
FOXp3	Log-rank 0.430	Log-rank 2.834	Log-rank 2.552	0/1st-T vs. 2nd-T/3rd-T

For the biomarkers that scored via immunohistochemistry (PARP, ATM, H2Ax, PTEN, FANC, PD-L1, and PD-L2) as 0, 1+, 2+, or 3+, we have modeled the tested biomarkers as the following 3 patterns: 0 vs. 1+/2+/3+; 0/1+ vs. 2+/3+; and 0, 1+, 2+ vs 3+. In each cutoff, Log-rank test was performed for overall survival, and the statistical values are displayed in the table. Among the 3 cutoffs tested, the cutoff demonstrating the largest statistical value was chosen for the further survival analysis. For the biomarkers that scored as cell counts (CD4, CD8, CD68, and FOXp3), the cell counts among expressed were trisected as 1-33%ile (1st-T), 34-66%ile (2nd-T), and 67-100%ile (3rd-T). We have modeled the tested biomarkers as the following 3 patterns: 0 vs 1st-T/2nd-T/3rd-T; 0/1st-T vs 2nd-T/3rd-T; and 0/1st-T/2nd-T vs 3rd-T. In each cutoff, Log-rank test was performed for overall survival, and the statistical values are displayed in the table. Among the 3 cutoffs tested, the cutoff demonstrating the largest statistical value was chosen for the further survival analysis.

Table S3. Correlation for pathological factors among high-grade serous carcinoma (n = 141).

	No.	p53	PARP	ATM	H2Ax	PTEN	FANC	CD4	CD8	CD68	FOXp3	PD-L1	PD-L2
p53	138		0.06	0.04	0.01	0.01	0.07	0.08	0.03	0.10	0.01	0.11	0.07
PARP	141	0.51		**0.41**	0.10	0.04	**0.33**	**0.22**	0.12	0.01	0.05	**0.53**	0.03
ATM	135	0.66	**<0.001**		0.15	0.16	**0.25**	0.04	**0.32**	**0.24**	**0.19**	**0.29**	0.11
H2Ax	136	0.96	0.25	0.09		**0.17**	0.10	0.01	0.01	0.04	0.03	0.09	0.11
PTEN	137	0.99	0.67	0.07	**0.048**		0.06	0.05	0.06	0.09	0.02	**0.20**	0.02
FANC	136	0.39	**<0.001**	**0.004**	0.23	0.51		0.07	0.12	0.02	0.02	**0.30**	0.02
CD4	136	0.34	**0.011**	0.68	0.98	0.55	0.39		**0.42**	**0.46**	**0.43**	0.07	0.03
CD8	134	0.74	0.16	**<0.001**	0.92	0.51	0.17	**<0.001**		**0.48**	**0.53**	0.15	0.16
CD68	131	0.26	0.93	**0.006**	0.66	0.28	0.84	**<0.001**	**<0.001**		**0.56**	0.16	**0.17**
FOXp3	135	0.96	0.58	**0.030**	0.73	0.81	0.80	**<0.001**	**<0.001**	**<0.001**		0.08	0.11
PD-L1	138	0.22	**<0.001**	**0.001**	0.31	**0.018**	**<0.001**	0.43	0.09	0.062	0.36		0.10
PD-L2	135	0.41	0.72	0.20	0.21	0.82	0.80	0.78	0.064	**0.048**	0.22	0.24	

Spearman's correlation coefficient for p-values (right upper half shows R values, and left lower half shows p-values). Significant p-values ($p < 0.05$) are emboldened.
